# Genome Wide Identification, Phylogeny, and Expression of *Aquaporin* Genes in Common Carp (Cyprinus carpio)

**DOI:** 10.1371/journal.pone.0166160

**Published:** 2016-12-09

**Authors:** Chuanju Dong, Lin Chen, Jingyan Feng, Jian Xu, Shahid Mahboob, Khalid Al-Ghanim, Xuejun Li, Peng Xu

**Affiliations:** 1 College of Fishery, Henan Normal University, Xinxiang, Henan, China; 2 College of Life Sciences, Shanghai Ocean University, Shanghai, China; 3 Fujian Collaborative Innovation Center for Exploitation and Utilization of Marine Biological Resources, College of Ocean and Earth Sciences, Xiamen University, Xiamen, China; 4 CAFS Key Laboratory of Aquatic Genomics and Beijing Key Laboratory of Fishery Biotechnology, Centre for Applied Aquatic Genomics, Chinese Academy of Fishery Sciences, Beijing, China; 5 Department of Zoology, College of Science, King Saud University, Riyadh, Saudi Arabia; 6 Department of Zoology, GC University, Faisalabad, Pakistan; Universidade de Lisboa Faculdade de Farmacia, PORTUGAL

## Abstract

**Background:**

Aquaporins (Aqps) are integral membrane proteins that facilitate the transport of water and small solutes across cell membranes. Among vertebrate species, Aqps are highly conserved in both gene structure and amino acid sequence. These proteins are vital for maintaining water homeostasis in living organisms, especially for aquatic animals such as teleost fish. Studies on teleost Aqps are mainly limited to several model species with diploid genomes. Common carp, which has a tetraploidized genome, is one of the most common aquaculture species being adapted to a wide range of aquatic environments. The complete common carp genome has recently been released, providing us the possibility for gene evolution of *aqp* gene family after whole genome duplication.

**Results:**

In this study, we identified a total of 37 *aqp* genes from common carp genome. Phylogenetic analysis revealed that most of *aqp*s are highly conserved. Comparative analysis was performed across five typical vertebrate genomes. We found that almost all of the *aqp* genes in common carp were duplicated in the evolution of the gene family. We postulated that the expansion of the *aqp* gene family in common carp was the result of an additional whole genome duplication event and that the *aqp* gene family in other teleosts has been lost in their evolution history with the reason that the functions of genes are redundant and conservation. Expression patterns were assessed in various tissues, including brain, heart, spleen, liver, intestine, gill, muscle, and skin, which demonstrated the comprehensive expression profiles of *aqp* genes in the tetraploidized genome. Significant gene expression divergences have been observed, revealing substantial expression divergences or functional divergences in those duplicated *aqp* genes post the latest WGD event.

**Conclusions:**

To some extent, the gene families are also considered as a unique source for evolutionary studies. Moreover, the whole set of common carp *aqp* gene family provides an essential genomic resource for future biochemical, toxicological, physiological, and evolutionary studies in common carp.

## Introduction

Aquaporins (Aqps) are a large superfamily of major intrinsic proteins (MIP), which selectively control the flow of water and other small molecules through biological membranes [1]. Therefore, Aqps play an important role in maintaining body osmotic balance for many organisms, especially for aquatic organisms. The first water channel protein was reported as Aqp1, which plays diverse roles in the mammalian erythrocytes [1, 2]. Since then, more and more genome-wide analyses of *aqp*s have been published, and deposited thousands of *aqp* genes into public databases. For instance, it was reported that zebrafish (*Danio rerio*) have up to 20 *aqp* genes [3] in the diploid genome. There are 42 *aqp* paralogs in the Atlantic salmon (*Salmo salar*) genome, which has experienced an additional round of whole genome duplication (WGD) compared with most other diploid teleost fish [3]. Therefore, the *aqp* genes are in the duplication manners in the tetraploidized salmon genome.

Vertebrate *aqp*s used to be classified into 13 classes. However, recent study has reported a total of 17 classes in *aqp*s gene family in various vertebrates by *Finn* et al. Besides of 13 *aqp* classes retained in human genome, there are four additional classes, including *aqp*13 in aquaglyceroporins, *aqp*14 and *aqp*15 in classical aquaporins, and *aqp*16 in aquaporin-8 in various vertebrate genomes. Therefore, the vertebrate *aqp*s were classified as follows: classical aquaporins (*aqp* 0, -1, -2, -4, -5, -6, -14, and -15), aquaglyceroporins (*aqp* 3, -7, -9, -10, and -13), aquaporin-8 (*aqp* 8, and -16), and unorthodox aquaporins (*aqp* 11 and -12) [3]. Although overall primary sequences are not well conserved (approximately 30% identity), all Aqps share a relatively conserved molecular structure, containing six membrane-spanning segments (TM1–TM6) with five connecting loops (LA–LE) [4]. Each Aqp half contains a conserved asparagine–proline–alanine (NPA) motif, located at LB and LE, that form short hydrophobic helices and dip halfway into the membrane from opposite sides, facing each other and participating in substrate selectivity [5]. A cysteine residue at position 189 in LE of human Aqp1 and 181 of Aqp2 is responsible for conferring mercury sensitivity [6]. In the biological membrane, Aqps are grouped as homotetramers embedded in the lipid bilayer and each monomer functions independently as a single pore channel [5].

Cyprinids are one of the most important teleost families in the world. Many species are domesticated as important aquaculture fish for food and ornamental purpose. Despite of the importance of Aqps for teleost fish, limited studies have been performed on *aqp* gene family in cyprinid species, except model species zebrafish. Common carp (*Cyprinus carpio*) originated in Europe and Asia. The species has been domesticated and introduced into various environments worldwide. It is an important economic and model species for various studies on ecology, environmental toxicology, developmental biology, nutrition, physiology, immunology, and evolutionary genomics. Therefore, significant genome resources have been developed in the past decade, including vast amount of genetic markers [7–10], genetic maps [11–13], BAC libraries and physical maps [14–16], expressed sequence tags (ESTs) [17], and transcriptome sequences [8, 18]. Recently, the common carp genome has been completely sequenced and assembled [19]. The evidence has shown that common carp is a species with an allotetraploidized genome, which had experienced an additional round of whole genome duplication (WGD). It has been hypothesized that the duplicated genome provides the basis for its enhanced adaptation to varied environments [20]. Therefore, it is of interest to determine if the number of *aqp* genes is doubled comparing with that of other diploid teleost fish, and elucidate Aqp functional evolution post the most recent WGD event.

In this study, by utilizing all available common carp genomic resources, we identified 37 *aqp* genes across the genome. Further phylogenetic analysis confirmed the gene annotation and nomenclature. Moreover, we examined the tissue distribution of *aqp* genes in common carp. The expression patterns of each gene, together with the results from comparative study with other vertebrate species, were used to infer the potential functions of *aqp* genes in common carp. Our study on examining the *aqp* gene family in common carp provides insights into the evolutionary and physiological aspects of post-WGD adaptation in common carp.

## Results and Discussion

### *Aqp* gene identification and characterization

We have identified a total of 20 *aqp* genes from zebrafish (*D*. *Rerio*) genome. Therefore, we used the 20 *aqp* genes of zebrafish as query to screen their orthologs in common carp genome. A total of 37 putative members of the *aqp* gene family have been identified from common carp genome. The 37 genes were distributed on 19 chromosomes and 18 scaffolds in the common carp genome, which are significantly more abundant than that in most other vertebrate genomes. For instance, there are 19 *aqp* genes in human (*H*. *Sapiens*), and 15 *aqp* genes in clawed frog (*X*. *Tropicalis*) and 12 *aqp* genes in medaka (*O*. *Latip*es) genome. Detailed information of their location, corresponding genomic sequences, coding sequences and DDBJ database accession number are summarized in Table 1.

**Table 1:** Summary of *aqp* family in the common carp genome.

Gene name	Location	CDS (na)	CDS (aa)	CDS status	Accession number
*aqp*0aa-1	Scaffold	792	263	Complete	LC069001
*aqp*0aa-2	Scaffold	792	263	Complete	LC069002
*aqp*0ab-1	Scaffold	837	278	Complete	LC068999
*aqp*0ab-2	Scaffold	795	264	Complete	LC069000
*aqp*1a-1	LG4	780	259	Complete	LC069004
*aqp*1a-2	LG2	783	260	Complete	LC069005
*aqp*1b-1	LG4	801	266	Complete	LC069006
*aqp*1b-2	LG4	810	269	Complete	LC069007
*aqp*3a-1	LG42	891	296	Complete	LC069008
*aqp*3a-2	LG3	891	296	Complete	LC069009
*aqp*3b-1	LG41	900	299	Complete	LC069010
*aqp*3b-2	LG41	897	298	Complete	LC069011
*aqp*4b-1	LG40	996	331	Complete	LC069003
*aqp*4b-2	Scaffold	996	331	Complete	LC149722
*aqp*7-1	Scaffold	912	303	Complete	LC069015
*aqp*7-2	Scaffold	825	218	Partial	LC177757
*aqp*8aa-1	LG6	606	201	Partial	LC069017
*aqp*8aa-2	LG32	783	260	Complete	LC069018
*aqp*8ab-1	LG23	774	257	Complete	LC069019
*aqp*8ab-2	LG23	279	166	Partial	LC149723
*aqp*8bb	LG6	777	258	Complete	LC069020
*aqp*9a-1	Scaffold	882	293	Complete	LC149726
*aqp*9a-2	Scaffold	654	217	Partial	LC149727
*aqp*9b-1	Scaffold	873	290	Complete	LC069012
*aqp*9b-2	LG40	873	290	Complete	LC069013
*aqp*10a-1	LG32	903	300	Partial	LC069014
*aqp*10a-2	Scaffold	944	313	Complete	LC177758
*aqp*10b-1	Scaffold	936	311	Complete	LC069016
*aqp*10b-2	Scaffold	987	328	Complete	LC177759
*aqp*11b-1	LG35	831	276	Complete	LC069021
*aqp*11b-2	Scaffold	837	278	Complete	LC069022
*aqp*12-1	Scaffold	843	280	Complete	LC069023
*aqp*12-2	LG44	831	276	Complete	LC069024
*aqp*14b-1	Scaffold	834	277	Complete	LC149724
*aqp*14b-2	LG40	819	272	Complete	LC149725
*aqp*15-1	Scaffold	1182	307	Partial	LC177760
*aqp*15-2	Scaffold	465	154	Partial	LC177761

The functional domains of *aqp* genes were predicted based on their protein sequences. As shown in S1 Fig, all Aqps possess one conserved domain (MIP) except for Aqp8ab-2, which is consistent with previous reports on Aqp protein structure [21], indicating high conservation of *aqp*s. Additionally, there are four Aqps (Aqp7-2, 11b-1, 11b-2 and 15–2) contain the transmembrane domain (TM) at the N-terminal or C-terminal end of the protein, and eight Aqps (Aqp3b-1, 3b-2, 4b-1, 8ab-2, 9a-1, 9a-2, 10a-1 and 10a-2) exhibited low complexity (LW) in the domain structure prediction.

### Phylogenetic analysis and nomenclature of *aqp* gene family in common carp

In the evolution of higher eukaryotes, WGDs followed by polyploidization, as well as gene loss, have been an important recurrent process. Ancient WGDs, inferred from analyzed sequenced genomes and comparative genomics, are prevalent and recurring throughout the evolutionary history of higher eukaryotic lineages [22]. To examine phylogenetic relationships of *aqp* genes in the teleosts and representative higher organisms, we collected a total of 103 *aqp* genes from five species, including human, clawed frog, medaka, zebrafish, and common carp. Also, phylogenetic analysis can be used to support the gene annotation, especially for non-model species [23], we investigated the molecular phylogeny of these *aqp* genes to validate the orthology of the common carp *aqp*s.

**Fig 1:**
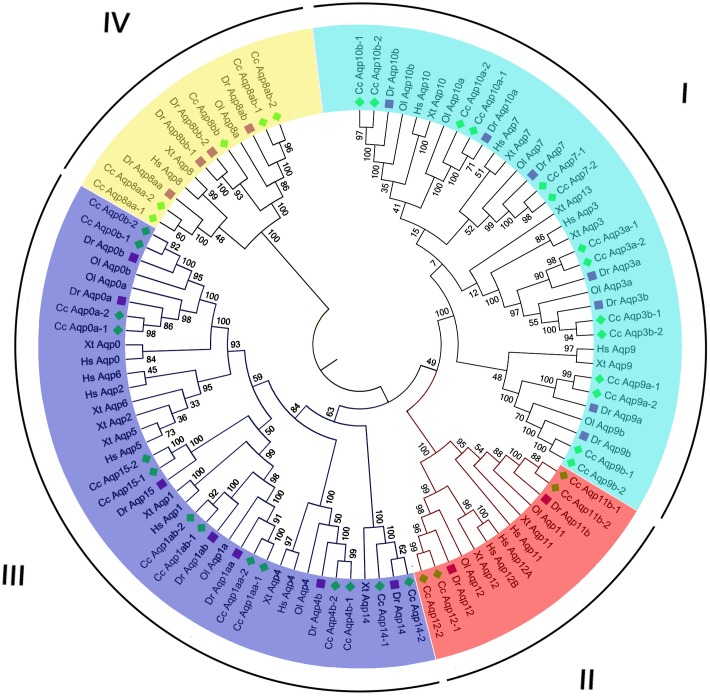
Neighbor-joining -based phylogenetic tree of 103 Aqp protein sequences. The *aqp* gene family is separated into four clades. The Aqp amino acid sequences are collected from the following vertebrates: human (Hs), zebrafish (Dr), medaka (Ol), frogs (Xt), and common carp (Cc).

Two phylogenetic dendrograms constructed based on alignments of the amino acid sequences of the Aqp proteins using both neighbor-joining (NJ, Fig 1) and maximum likelihood (ML, S2 Fig) showed high topological consistency, indicating the reliability of the phylogenetic relationships of the *aqp* genes. As shown in these two figures, the phylogenetic analysis results showed that each of common carp *aqp* genes clustered with its respective counterpart from other species, indicating all genes are highly conserved.

For *aqp* gene family, as revealed by phylogenetic tree, all of the *aqps* were categorized into four clades. Also, we speculated that these genes falling into one clade may be derived from a very ancient lineage of vertebrate. Similar relationships of these genes have been reported in mammalian genes, which therefore are divided into four subfamilies [24]. The 103 vertebrate Aqps formed four distinct monophyletic groups orresponding to four subfamilies. The four subfamilies were characterized based on the topology: subfamily I (classical aquaporins), which contained Aqp 0, 1, 2, 4, 5, 6, 14 and 15; subfamily II (unorthodox aquaporins), which contained Aqp 11, and 12; subfamily III (aquaglyceroporin) which contained Aqp 3, 7, 9 and 10; and subfamily IV (aquaporin-8), which contained Aqp8.

The nomenclature of these genes was based on their identity to zebrafish orthologs and their phylogenetic position. Additionally, all the Aqps of common carp were categorized into a teleost subclade with members of the medaka and zebrafish. We therefore annotated each of the common carp paralogs with the postscript “-1” or “-2” following the name of zebrafish orthologs to reflect the 4R WGD [3, 25]. According to the naming strategy, none of *aqp* genes in common carp were presented as single copy, while *aqp*4, 7, 11, 12, 14 and 15 (Cc *aqp*4b-1, 4b-2, 7–1, 7–2, 11b-1, 11b-2, 12–1, 12–2, 14–1, 14–2, 15–1 and 15–2) have two copies, *aqp*0, 1, 3, -9 and 10 (Cc *aqp*0a-1, 0a-2, 0b-1, -0b-2, 1aa-1, 1aa-2, 1ab-1, 1ab-2, 3a-1, 3a-2, 3b-1, 3b-2, 9a-1, 9a-2, 9b-1, 9b-2, 10a-1, 10a-2, 10b-1 and 10b-2) have four copies, and *aqp*8 (Cc *aqp*8aa-1, 8aa-2, 8ab-1, 8ab-2, 8bb) has five copies, respectively (Table 2).

**Table 2:** *aqp* gene family in the genomes of the five vertebrates.

*C*.*carpio*	*D*.*rerio*	*O*.*latipes*	*X*.*tropicalis*	*H*.*sapiens*
37	20	12	15	19
*aqp*0a-1	*aqp*0a	*aqp*0a	*aqp*0	*aqp*0
*aqp*0a-2				
*aqp*0b-1	*aqp*0b	*aqp*0b		
*aqp*0b-2				
*aqp*1aa-1	*aqp*1aa	*aqp*1a	*aqp*1	*aqp*1
*aqp*1aa-2				
*aqp*1ab-1	*aqp*1ab			
*aqp*1ab-2			*aqp*2	*aqp*2
*aqp*3a-1	*aqp*3a	*aqp*3a	*aqp*3	*aqp*3
*aqp*3a-2				
*aqp*3b-1	*aqp*3b			
*aqp*3b-2				
*aqp*4b-1	*aqp*4b	*aqp*4	*aqp*4	*aqp*4
*aqp*4b-2				
			*aqp*5	*aqp*5
			*aqp*6	*aqp*6
*aqp*7a	*aqp*7	*aqp*7	*aqp*7	*aqp* 7
*aqp*7b				
*aqp*8aa-1	*aqp*8aa	*aqp*8a	*aqp*8	*aqp*8
*aqp*8aa-2	*aqp*8ab			
*aqp*8ab-1	*aqp*8bb-1			
*aqp*8ab-2	*aqp*8bb-2			
*aqp*8bb				
*aqp*9a-1	*aqp*9a		*aqp*9	*aqp*9
*aqp*9a-2				
*aqp*9b-1	*aqp*9b	*aqp*9b		
*aqp*9b-2				
*aqp*10a-1	*aqp*10a	*aqp*10a	*aqp*10	*aqp*10
*aqp*10a-2				
*aqp*10b-1	*aqp*10b	*aqp*10b		
*aqp*10b-2				
*aqp*11b-1	*aqp*11b	*aqp*11	*aqp*11	*aqp*11
*aqp*11b-2				
*aqp*12-1	*aqp*12	*aqp*12	*aqp*12	*aqp*12A
*aqp*12-2				*aqp*12B
			*aqp*13	
*aqp*14-1	*aqp*14		*aqp*14	
*aqp*14-2				
*aqp*15-1	*aqp*15			
*aqp*15-2				

As previous studies reported, two rounds of WGD have occurred in the ancestor of vertebrates, plus two in the lineage of common carp, of which, the 3R WGD is known as teleost-specific (TS) WGD, and the 4R WGD is only occurred in some tetraploid teleost, such as salmonids and some cyprinids [26]. Common carp genome had been previously confirmed as allotetraploidized genome based on comparative genomic studies [19]. Significant gene duplications are presented in the Aqp topologies of common carp, which are clearly consistent with previous findings [27–29].

### Gene duplications and losses in common carp

WGD is one of the major drivers that shaped the evolutionary history of many vertebrates. Ohno has suggested that two rounds of large-scale gene duplication had occurred early in vertebrate evolution [30], and a number of studies of comparative analysis of various gene clusters provided solid evidence in support of Ohno's hypothesis [31–33]. An additional round of duplication, also named teleost-specific (TS) WGD, or the 3R WGD [34, 35], took place in the common ancestor of all extant teleosts.

As a result of genome duplication, teleost fish usually have two paralogous copies for many genes, while only one ortholog is present in tetrapods. Also, it is generally-accepted hypothesized that, comparing to other teleost, salmonids and some cyprinids such as common carp and goldfish had undergone additional whole genome duplication (the 4R WGD) [27], Microsatellite analysis [28] and comparing common carp linkage map to zebrafish genome [29] provided critical evidence in support of the 4R WGD event in common carp [36]. The comprehensive estimation based on whole genome datasets suggests that the latest WGD event occurred around 8.2 MYA [37]. Therefore, the significant expansion of *aqp* genes in the common carp genome may be the result of this additional WGD, which could have caused a sudden doubling of the *aqp* genes. As shown in Table 2, common carp retained double or more than double the aqp copies of the zebrafish *aqp* genes, except aqp8bb, which strongly suggests that the 4RWGD event was the major contributor to *aqp* gene family expansion in common carp. Similar results were observed when to the common carp *aqp* genes were compared with the *aqp* genes in other teleost genomes.

**Fig 2:**
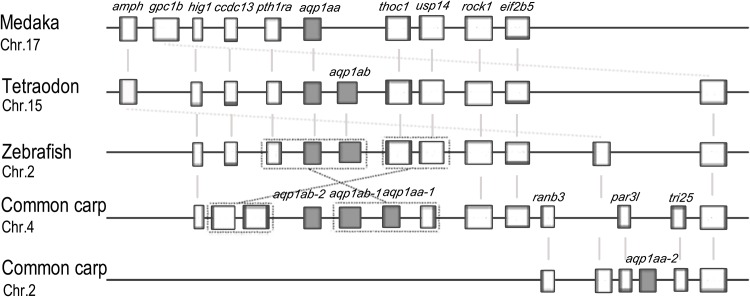
Conserved synteny blocks harboring the *aqp*1s in vertebrates. Vertical lines denote orthologous relationships.

Although mammals and teleosts last shared a common ancestor many hundred million years ago, a growing number of studies have reported extensive conserved synteny between the chromosomes of teleosts and mammals, which favors the rule of additional genome duplication in fishes [38]. In this study, syntenic blocks of *aqp*1 genes were constructed as shown in Fig 2. Clearly, in zebrafish, the two *aqp* genes are on chromosomes 2 and, in common carp, the four genes were distribute on chromosomes 2 and 4. Here, we consider a new evolutionary scenario to explain the gene duplication event in common carp. Assuming that the putative teleost ancestor had the *aqp*1 aa/ab genes on two different chromosomes, then, when genome duplication was finished in common carp, the four orthologs (*aqp*1 aa-1/aa-2/ab-1/ab-2) were distributed on four different chromosomes. However, with the cis and trans mechanisms, in the common carp genome, the two *aqp* genes on other chromosomes moved to chromosome 4, resulting in three *aqp* genes on chromosome 2 and one *aqp* on chromosome 4 (Fig 2). This hypothesis can explain the distribution of *aqp* in the common carp genome reasonably.

After duplication, one of the two redundant copies of a gene should theoretically be free to degenerate and become lost from the genome without consequence [39, 40]. Most gene pairs formed by a WGD have only a brief lifespan before one copy becomes deleted, leaving the other to survive as a single-copy locus. We observed that there are only one *aqp*8bb in common carp and two copies in zebrafish, which is different with other gene number comparison. The Aqp8bb protein sequences were found to be highly conserved across all the vertebrate species, suggesting that the conserved *aqp*8bb gene is critical for survivability and very little change is allowed in its coding sequence and copy number in common carp. Abundant copies of a single gene might accumulate detrimental mutations due to relaxed selection on one of the duplicates. Gradually, they will become pseudogenized and they will either be deleted from the genome or become so diverged from the parental genes that they are not identifiable any longer [41, 42]. In addition, we have not identified that *aqp* 2, 5, and 6 are absence in all surveyed teleost fish but retained in other vertebrates, which is consistent with previous report [3], suggests the gene losses occurred in the common ancestor of teleost fish post the divergence of teleost fish and tetrapods. Regarding gene losses, it may occur in *aqp* gene family in common carp post the latest WGD as those identified 37 *aqp* genes are much less than our expectation, however, we also suspect another possibility that imperfect genome assembly and annotation lead to the “gene losses”, especially on such a tetraploidized genome of common carp [43].

### Expression profiling of *aqp* genes in common carp and potential functional inferences

**Fig 3:**
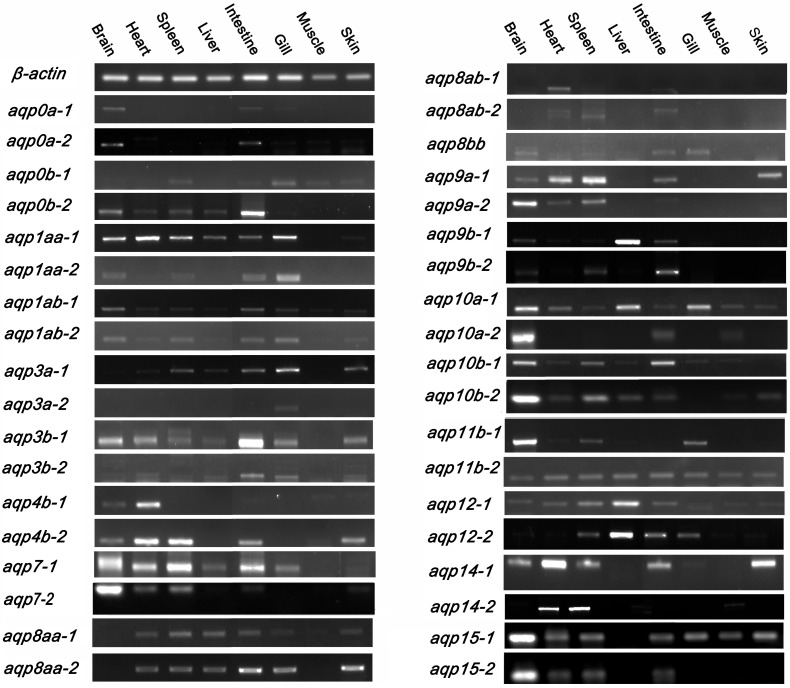
RT-PCR based expression analysis of *aqp* genes in eight tissues of common carp. β-actin was used as an internal control, gene names are indicated on the left of the panel. The eight tissues are brain, heart, spleen, liver, intestine, muscle, gill and skin.

Exploring expression profiling of *aqp*s could help to speculate their functions. The relative expression of the common carp *aqp* genes in adult tissues was evaluated by RT-PCR employing isoform-specific oligonucleotide primers. As shown in Fig 3, the *aqp* gene family exhibited unique tissue-specific expression. In general, most of the *aqp* genes were widely expressed, but has a relatively high expression levels in brain, spleen and intestine and relative irregularity expression levels in other tissues. Also, we observed that *aqp* genes were almost no expressed in muscle, implying their unimportant roles in muscle organ development. *Aqp*8aa-1 and *aqp*14-1 were highly expressed in skin, suggesting their specific expression and special functions in the development of skin in common carp. As expected, we do observed significant difference on *aqp* expression profiles (Fig 3), which implied the functional divergence of duplicated *aqp* genes. For instance, we observed some consistent expression patterns in two copies of *aqp* genes in common carp, including *aqp* 0a, 1ab, 3b and *aqp* 8aa. Moreover, distinct expression patterns in two copies of 15 *aqp* genes, including *aqp* 0b, 1aa, 3a, 4b, 7, 8ab, 9a, 9b, 10a, 10b, 11b, 12, 14 and 15 were also obsevered. The two copies of *aqp* 0b exhibit an almost complementary expression pattern. For the rest of the 14 *aqp* genes, one of the two copies have broad expression profiles in surveyed tissues and the other one have relatively narrow expression. The spatial expression difference of the two copies of genes suggested quick functional divergence of these newly emerged *aqp* copies. It has been recommended that unless the presence of an extra gene product is of advantage, two genes with identical functions are unlikely to be stably maintained in the genome [42]. As the results, the duplicates would develop difference in some functional aspect, such as subfunctionalization, which could be stablely maintained in the genome. The expression profiles of *aqp* gene family in common carp suggested that *aqp* duplicates evolve quickly and subfunctionalization is commonly occurred in the tetraploidized genome.

We also observed significant gene expression differences compared with previous studies on model species. For instance, comparing the expression patterns with other vertebrate species, like zebrafish and human, conservation/divergence patterns were revealed as expected. In common carp, three of the four *aqp*1 copies showed tissue-wide expression patterns, while the remaining *aqp*1aa-2 had a tissue-specific expression pattern. Similar cases occurred in zebrafish, where one of the *aqp*1 genes the most ubiquitously expressed *aqps*, while another *aqp* otholog only expressed in several specific tissues [44], consistent with the presence of the human *aqp*1 ortholog [45]. Furthermore, *aqp*3 are mostly ubiquitously expressed in human and common carp tissues, however, there are no gene expression in the liver of zebrafish which are different with its orthologs in both common carp and human. These phenomenon maybe indicated that the expression pattern in different genes is different and has its own species-specific [46]. Obviously, these significant expression differences in those duplicated *aqp* genes, providing evidence for gene subfunctionalization post-WGD event. Most likely, the ancestral gene was capable of performing all functions and was expressed broadly in the tissues, while the descendant duplicate genes only perform partial functions and are specifically expressed in certain tissues. The functional divergence of duplicated genes may avoid potential adaptive conflicts [45].

## Conclusion

In this study, we identified a total of 37 *aqp* genes in tetraploidized common carp genome. Phylogenetic and syntenic analysis as well as comparative genomic study revealed comprehensive understanding of *aqp* gene family and their distribution in the genome. Our analyses revealed extensive gene duplications in common carp which result from the additional WGD in common carp. Expression profiles of the complete set of *aqp* genes in common carp were assessed, which revealed extensive gene functional divergence in *aqp*s in common carp. Our study provides essential genomic resources for future biochemical, toxicological, physiological, and evolutionary studies in common carp.

## Materials and Methods

### Ethic statement

This study was approved by the Animal Care and Use committee of Centre for Applied Aquatic Genomics at Chinese Academy of Fishery Sciences. The methods were carried out in accordance with approved guidelines. Adult common carp were collected from the Breeding Station of Henan Academy of Fishery Research, Zhengzhou, Henan province, China. Euthanasia is performed by immersion fish in MS-222 solution, and all efforts were made to minimize suffering.

### Aqp identification and sequence analysis

All available *aqp* gene sequences and Aqp amino acid sequence from four species (human, clawed frog, medaka, zebrafish) were downloaded from public database Ensembl (http://asia.ensembl.org/), GenBank (http://www.ncbi.nlm.nih.gov/genbank/) and ZFIN (http://zfin.org/). The genomes of these four species have been well-characterized and annotated previously. Amino acid sequences of Aqps in zebrafish were used as queries to search against all available common carp genomic resources by BLAST tools, with an *E*-value cutoff of 1e-5 to acquire the candidate *aqp* genes. Then reciprocal BLAST searches were conducted by using the candidate common carp *aqp* genes as queries to verify the veracity of candidate genes. The predicted sequences were extracted, analyzed, and confirmed by BLASTP searches against the NCBI non-redundant protein sequence database (nr).

The simple modular architecture research tool (SMART) was used to predict the conserved domains in common carp AQPs. The simple modular architecture research tool (SMART, http://smart.embl-heidelberg.de/) was used to predict the conserved domains based on sequence homology and further confirmed by “conserved domains” prediction software (http://www.ncbi.nlm.nih.gov/Structure/cdd/wrpsb.cgi?) in NCBI.

### Phylogenetic analysis

To annotate the *aqp* genes, phylogenetic analysis was conducted with reference Aqp proteins from zebrafish, and other representative vertebrate species. First of all, the Aqp protein sequences of four surveyed species were downloaded from the Ensemble databases. Then, the translated protein sequences of the common carp *aqp* orthologous genes and the Aqp protein sequences from the four other species (a total of 103 sequences) were aligned using ClustalW2 (http://www.ebi.ac.uk/Tools/msa/clustalw2/). The sequences were then manually trimmed of all sites that were not unambiguously aligned. The deduced Aqp protein sequences were used for phylogenetic analysis in conjunction with reference Aqp proteins from zebrafish, medaka, frog, and human. We performed neighbor-joining (NJ) analysis in MEGA7 [47] with the p-distance model/method. Also, a maximum likelihood (ML) tree with default parameters was constructed using the MEGA7 to verify the accuracy of the toplogy of NJ tree. A total of 1000 bootstrap replicates were conducted for each calculation.

### Gene nomenclature

Zebrafish *aqp* genes were named in accordance with previous studies [44, 48]. The *aqp* orthologous genes in common carp were named based on their phylogenetic topologies as well as BLAST result with their most related zebrafish genes. First, the subfamilies and gene members were determined for each common carp *aqp* orthologs based on the phylogenetic clades and the result of BLAST (for instance, *aqp* 0, *aqp* 1, etc.). Then, the closely related zebrafish *aqp* genes were assigned to each common carp *aqp* ortholog and the *aqp* genes were named after their most closely related zebrafish gene. When more than one copy of a common carp *aqp* gene was clustered with a certain zebrafish *aqp* gene, latin numbers suffixes were added to each copy (for instance, *aqp* 0a1, *aqp* 0a2, *aqp* 0b1, *aqp* 0b2, etc.). The names of each *aqp* gene in common carp and other surveyed species are listed in Tables 1 and 2.

### Syntenic analysis

Syntenic analyses were performed on selected *aqp* genes across the human, zebrafish, and common carp chromosomes by identifying the positions of *aqp* neighboring genes. The organization of the genes on the chromosomes of the model species was obtained from the Ensemble databases, while the gene organization of common carp was based on the draft sequences of the common carp genome assembly. Syntenic maps were then drawn based on the gene locations in the surveyed species.

### Expression profiling of *aqp* genes

Total RNA from various adult common carp tissues (brain, heart, spleen, liver, intestine, gill, muscle, and skin) was extracted with TRIzol^®^ reagent (Life Technologies, NY, USA). The cDNA, which was used for PCR to examine the *aqp* expression patterns, was synthesized by RT-PCR using the SuperScript^®^ III Synthesis System (Life Technologies). The β-actin gene was used as an internal positive control, with forward primer (5′ 9-TGCAAAGCCGGATTCGCTGG-3′ 9) and reverse primer (5′ 9-AGTTGGTGACAATACCGTGC-3′ 9). The whole PCR process was designed as follows: denaturation step for 5min at 94°C, 35cycles of denaturation (30 sec at 94°C), annealing (30 s), the temperature of which differed according various primers, and extension (30s at 72°C), and a final elongation step of 5 min at 72°C. The PCR products were separated by gel electrophoresis (1.5% agarose gel at 150 V) in the presence of ethidium bromide and visualized under ultraviolet light.

## Supporting Information

S1 Fig Schematic of the domain architecture of Aqp protein in common carp. (JPG)

S2 Fig Maximum-likelihood-based phylogenetic tree of 103 Aqp protein sequences. The Aqp gene family is separated into four clades. The Aqp amino acid sequences are collected from the following vertebrates: human (Hs), zebrafish (Dr), medaka (Ol), frogs (Xt), and common carp (Cc).
(JPG)

S1 Table Protein sequences of Aqps in common carp. (DOCX)
