# Supplementary figures and images for: Genome Wide Identification, Phylogeny, and Expression of Aquaporin Genes in Common Carp (Cyprinus carpio)
Source: PLoS One. 2016 Dec 9;11(12):e0166160. doi: 10.1371/journal.pone.0166160 (PMC5147823; doi:10.1371/journal.pone.0166160)

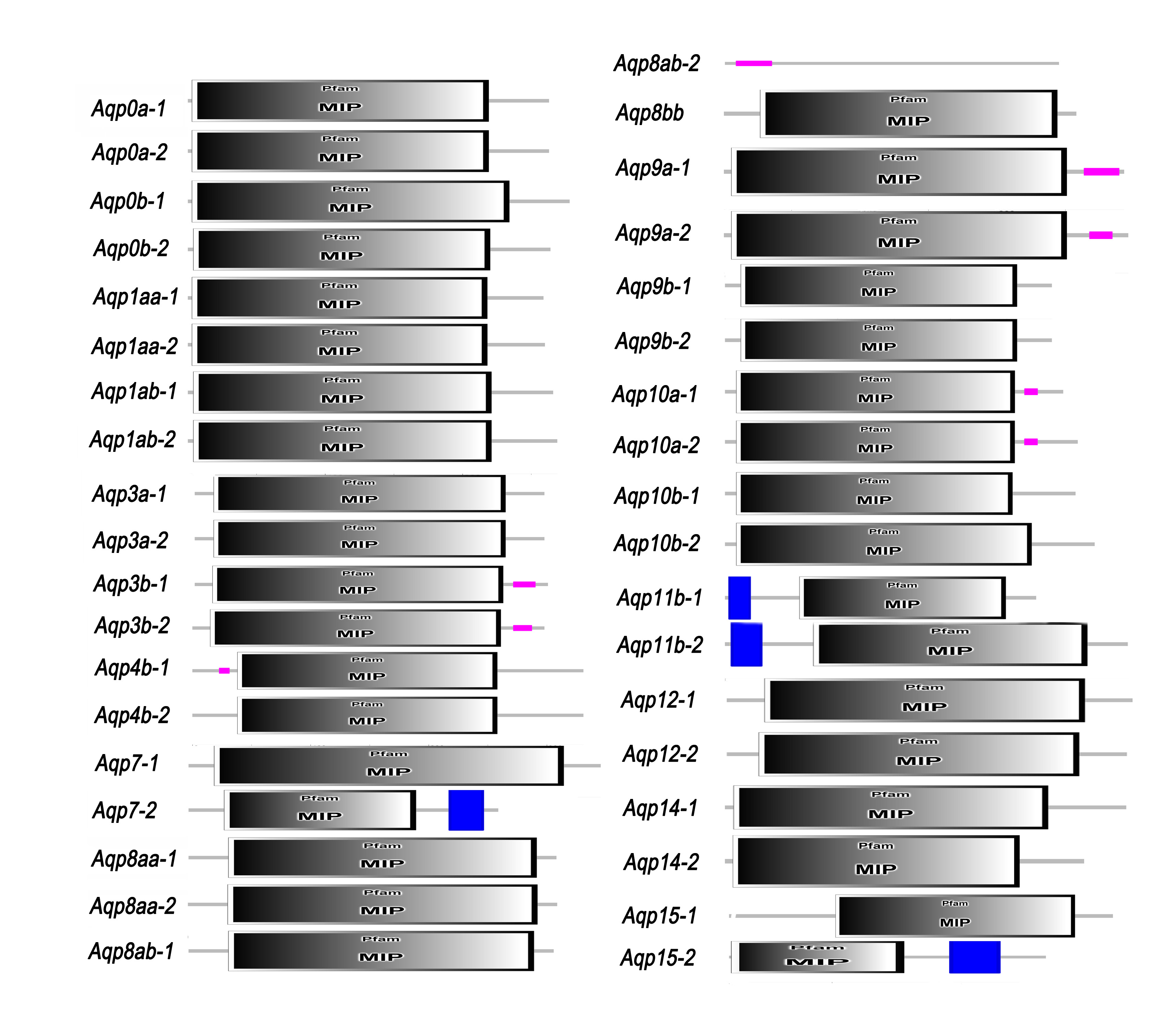

Supplement: S1 Fig — (JPG) [file pone.0166160.s001.jpg]

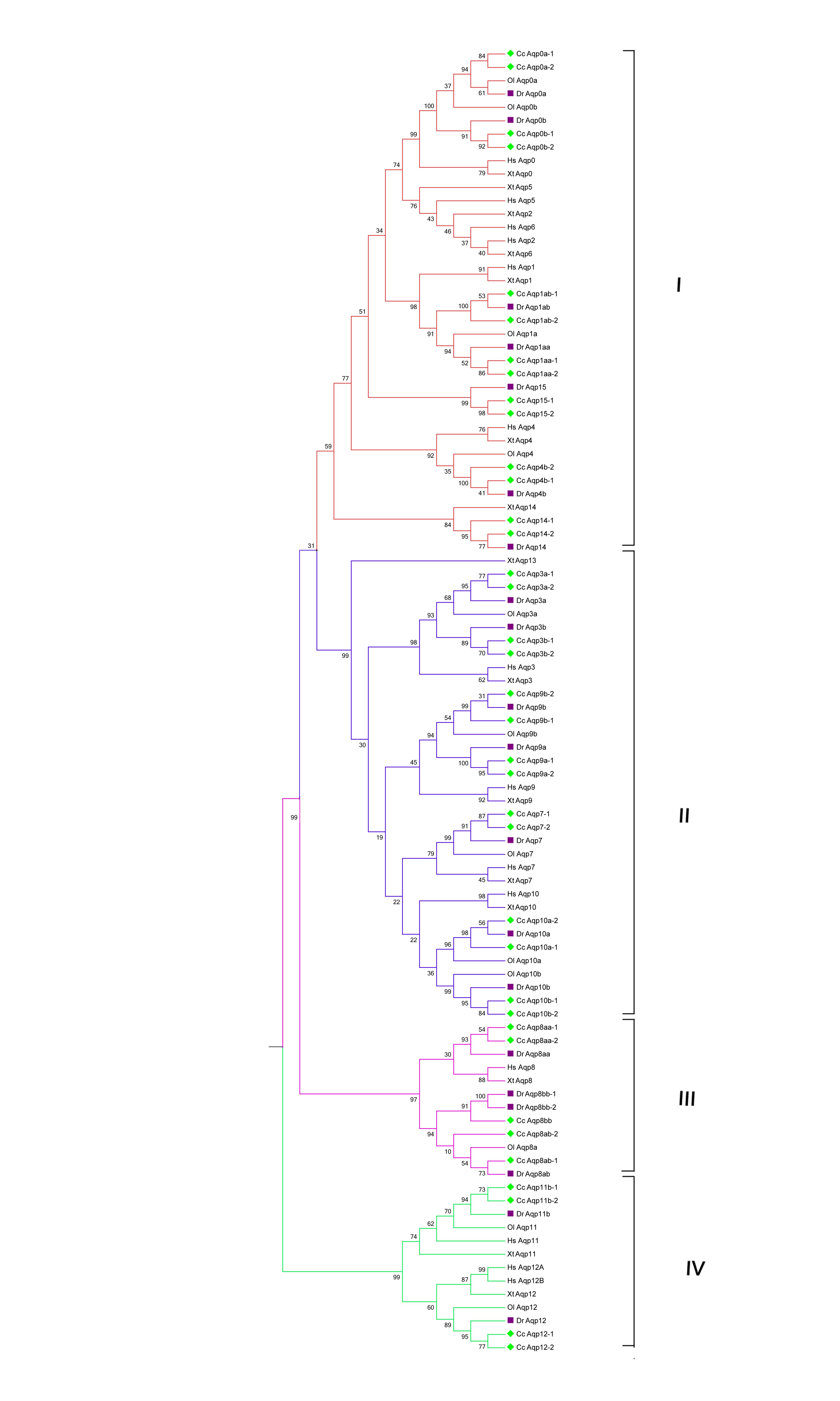

Supplement: S2 Fig — The Aqp gene family is separated into four clades. The Aqp amino acid sequences are collected from the following vertebrates: human (Hs), zebrafish (Dr), medaka (Ol), frogs (Xt), and common carp (Cc). (JPG) [file pone.0166160.s002.jpg]
