# Supplementary material for: Genome Wide Identification, Phylogeny, and Expression of Aquaporin Genes in Common Carp (Cyprinus carpio)
Source: PLoS One. 2016 Dec 9;11(12):e0166160. doi: 10.1371/journal.pone.0166160 (PMC5147823; doi:10.1371/journal.pone.0166160)
Supplement: S1 Table — (DOCX) [file pone.0166160.s003.docx]

**S1 Table. Protein sequences of Aqps in common carp.**

| **Gene name** | **Protein Sequence** |
| --- | --- |
| CcAqp0a-1 | MWEFRSMSFWRAVFAEFYGTMFFVFFGLGSALRWTTGPHNVLQVAFCFGLAAATLIQSIGHISGGHINPAVTFAYLISSQMSLFRAFFYICAQCFGALAGAGVLYGVTPNNMRGNLGLNTLQPGISVGMATTIEIFLTLQLVVVVFAVTDERRNGRLGSAALSIGFSVLVGHLLGMYYTGAGMNPARSFAPAVLFRNFINHWVYWVGPMIGGAMGALLYDFMLFPRMRDLSERLAVLKGNRPPEAEAQQETRGEPIELKTQAL |
| CcAqp0a-2 | MWEFRSMSFWRAVFAEFYGTMFFVFFGLGSALRWTTGPHNVLQVAFCFGLAAATLIQSIGHISGGHINPAVTFAYLIGSQMSLFRAFFYICAQCLGALAGAAVLYGVTPSNMRGNLGLNTLQPGISLGMATTIEIFLTLQLVVVVFAVTDERRNGRLGSAALSIGFSVLVGHLLGMYYTGAGMNPARSFAPAVLFRNFINHWVYWVGPMIGSAMGALLYDFMLFPRMRGLSERLAVLKGNRPPEAEAQQENRGEPIELKTQAL |
| CcAqp0b-1 | MWEFRSMMFWRAVFAEFFGTMFFVFFGMGAALRWTSGPYHVFHTALCFERSSTSALSDTALCFGFAAATLIQSIGHISGGHINPAVTFAYLVGSQMSFFRAFFYICAQCLGAMAGAAALYGVTPNNMRGTLALNTLQPGMSLGMATTVEVFLTMQLVVCVFAVTDERRNGRLGSAALSIGFSITMGHLMGMYYTGAGMNPARSFAPAIIMRNFINHWVYWVGPMIGGAMGAILYDFMLFPRMRGLSERLATLKGSRPPEAENQQETRGEPIELKTQTL |
| CcAqp0b-2 | MMWEFRSMMFWRAVFAEFFGTMFFVFFGMGAALRWTTGPYHVFHTALCFGFAAATLIQSIGHISGGHINPAVTFAYLVGSQMSFFRAFFYICAQCLGAMAGAAALYGVTPNNMRGTLALNTLQPGMSLGMATTVEVFLTLQLVVCVFAVTDERRSGRLGSAALSIGFSITMGHLMGMYYTGAGMNPARSFAPAIIMRNFINHWVYWVGPMIGGAMGAILYDFILFPRMRGLSERLATLKGSRPPEADNQQETRGEPIELKTQTL |
| CcAqp1aa-1 | MKELKSKAFWRAVLAELVGMTLFIFLSITAAVGNSNNDKPDQEVKVALAFGLSIATLAQSLGHISGAHLNPAVTLGLLASCQISLLKAVMYIVAQMFGAALASGIVYGVTDGNALGLNTISSKISPGQGVGIELFATFQLVLCVIATTDKRRRDVTGSAPLAIGLSVGLGHLTAISFTGCGINPARSFGPAVILRDFTNHWVYWVGPMCGGVAAALVYDFLLYPKLDDFPDRVRVLVSGPATDYDVNTDDPPAVEMSSK |
| CcAqp1aa-2 | MKELKSKAFWRAVLAELVGMTLFIFLSITAAVGNSNNDKPDQEVKVALAFGLSIATLAQSLGHISGAHLNPAVTLGLLASCQISLLKAVMYIVAQMFGAALASGIVYGVTDGKALGLNSISNSISPGQGVGIELFATFQLVLCVIATTDKKRRDVTGSAPLAIGLSVCLGHLTAISFTGCGINPARSFGPAVVLQDFTNHWVYWVGPMCGGVAAALVYDFLLYPKLDDFPDRVRVLVSGPATDYDLNVTNDPRAVEMSAK |
| CcAqp1ab-1 | MARELKSWSFWRAVLAEFVGMTVFVFIGIASAIGNKHNRYPDQEIKVALAFGLAIATLAQSLGHISGAHLNPAITLGLLVSCQISFFRASMYIVAQMLGAVLASGIMFKVSPDPESTLGLNMLSNGVKAGQGFAIELFATFQLVLCVLATTDKNRTDVTGSAPLAIGLSVGLGHLVAISYTGCGINPARSFGPAVILEAFKNHWIYWIAPMSGGVAAALVYDFLLCPKKEALRKRMNVLKGTADPDPSATEALIEPRTPRSGSGVE |
| CcAqp1ab-2 | MARELKSWSFWRAVLAEFVGMTVFVFIGIASAIGNKHNRYPDQEVKVALAFGLAIATLAQSLGHISGAHLNPAITLGLLVSCQISFFRAFMYIVAQMLGAVLASGIMFKVSPDPESTLGLNMLSNGVKPGQGFAIELFATFQLVLCVLATTDKHRTDVTGSAPLAIGLSVGLGHLVAISYTGCGINPARSFGPAVVLEAFKNHWIYWIAPMCGGVAAALVYDFLLCPKKEALRKRMNVLKGTADPDSSTTEPLIEPRTPRSGSGQWPRP |
| CcAqp3a-1 | MGWQKAMLDKLAHTFQIRNKLLRQGLAECLGTLILVMFGCGAVAQMVLSEKSHGRFLSVNLAFGFAATLGILVCGQVSGGHLNPAVTFALCLLGREKWRKFPVYFLFQTIGAFLGAAIIFAEYHDAMYEYAGEKNELLVTGDKATAGIFATYPGPHLTILNGFFDQVIGTAALIVCILAIVDPYNNPIPQGLEAFTVGFSVLIIGLSMGFNSGYAINPARDFGPRLFTSMAGWGGEVFTNRQCWFLVPMFAPFLGSIIGVLVYQLMVGWHVEGEVRDNKNKAKEESLKLNDVTSKD |
| CcAqp3a-2 | MGWQKDMLEKLAHTFQIRNKLLRQGLAECLGTLILVMFGCGAVAQMVLSRETHGRFLSVNLAFGFAATLGILVCGQVSGGHLNPAVTFALCLLGREKWRKFPVYFMFQTLGAFLGAAIIFAEYHDAMYDYSGENNVLLVTGENATAGIFATYPNPHLTILNGFFDQVIGTTSLIVCILAIVDPYNNPIPQGLEAFTVGFSVLIIGLSMGFNSGYAVNPARDFGPRLFTAMAGWGGEVFTTRECWFLVPFFAPFLGSIIGVIVYQLMVGWHVEGEVKNNKNKAREETLKLNDITSKE |
| CcAqp3b-1 | MGRQKVILEKMARIFHVRNVLIRQALAECLGTLILVMFGCGAVAQLILSGGSHGMFLTVNFAFGFAATLGILVCGQVSGGHINPTVTFSLCLLGREPWRKFPVFFLAQTLGAFLGSGIIFGMYIDAIWDYGQGSLVVVGDNATAGIFATYPSKHLTLLNGFFDQMIGTAALIVCILAIVDPYNNPIPQGLEAFTVGFVVLVIGQSMGFNSGYAVNPARDLGPRLFTALAGWGSEVFSANSYWSFVPIFAPFIGAMLGVMVYQLMVGYHVEGEARDKREAEEREEKERLKLSSVSEKETA |
| CcAqp3b-2 | MGRQKAILEKMARIFQVRNVLIRQALAECLGTLILVMFGCGSVAQLILSRGSHGMFLTVNFAFGFAATLGILVCGQVSGGHINPTVTFALCLLGREPWRKFPVYFLAQTLGAFLGSGIIFGMYFDAIWDYGQGSLVVVGDNATAGIFATYPSKHLTLLNGFFDQMIGTAALIVCILAIVDPYNNPIPQGLEAFTVGFVVLVIGQSMGFNSGYAVNPARDLGPRIFTAIAGWGSEVFSANSYWSFVPIFAPFIGAMLGVMVYQLMVGYHVEGEARDKRETEEKEKERFSLSAVSEKETP |
| CcAqp4b-1 | MRKALSQVSVPNRSCGFLRRCVSSCSCNNSIMAAFKGVWTQEFWRAVSGEFLAMIIFVLLSLGSTINWAATQENPPPPDLVLISLCFGLSIATLVQCFGHISGAHINPAVTVAMVATRKLSLAKGVFYLLAQCLGAVVGAAVLYGVTPASVRGGMGVTSVNAEISTGHAIVIELIITFELVFTVFATCDPKRNDLKGSAALAIGLSVCIGHLFAIPYTGASMNPARSFGPAVIMAKWQDHWVYWVGPLIGGILAAAVYEYLFCPDPDLKRRYADVLSKSHFQIDPYRVVDTDSYPSDQAQLMAKQAAIRVLDLERTQKKERESAGEVLSSV |
| CcAqp4b-2 | MRKAFSLVSVPNRSCEFLRRCGSSCSCNNTIMAAFKGVWTQEFWRAVSGEFLAMIIFVLLSLGSTINWAATQENPPPADLVLISLCFGLSIATLVQCFGHISGAHINPAVTTAMVATRKLSLAKGVFYLLAQCLGAVVGAAILYGVTPASVRGGMGVTSVNAEISTGHAIVIELIITFELVFTVFATCDPKRKDLKGSAALAIGLSVCIGHLFAIPYTGASMNPARSFGPAVIMAKWQDHWVYWVGPLIGGILAAAVYEYLFCPDPDLKRRYADVLSKSHFQIDPYRVVDTDSYPSDQAQLMAKQAAIRVLDLERAEKKERELAGEVLSSV |
| CcAqp7-1 | MEDVRIQDHMAPTEVFRLKIKNECIRVALAETLCTFIMMVFGLGTVAQVVTGNGVFGEYFSINVGFGLSVAMGVHVGGKVSGAHMNAAVSFTMCVFGRLRWKMFPLYVFAQFLGSFLAAGTIFLLYYDAIHHYCGGNFTVSGPKATAGIFATYPAPYISIYTGFFDQVLGTAMLLLCLMALADQRNQPVMSGGEPVGVGLLVLLIGVSLGSNSGYAINPTRDLGPRVFTLMAGWGMEVFRAGNGWWWVPLVAPFIGGVTGALIYEAFVELHHPDLKRTKTQSAEDPECVPLEKCKNGSTEMSV |
| CcAqp7-2 | MKQEVSIMEDVRIQDHMAPTEVFRLKIKNECIRVALAETLCTFIMMVFGLGTVAQVVTGNGVFGEYFSINVGFGLSVAMGVHVGGKVSGAHMNAAVSFTMCVFGRLRWKMFPLYVFAQFLGSFLAAGTIFLLSVTRKKINLSTTLELGHDLLITIISYQICKQEKNNLIQVLSHLIYRAGNGWWWVPLVAPFIGGVTGALIYEAFVELHHPDLKRTKT |
| CcAqp8aa-1 | MTSAESKSELFTVATGDGGDAHQNQPKLSFYEHYIQPCLAEVLGSTLFMFVGCVSVMGNVGISGSIQPALAHGLALAIAIAIFGEISGGHFNPAVSLCVYLIGGMELMLLVPYVLSQMLGGVIAASLAKAVTTDEAFGNATGAAFGAVQSSNGIGSATMAEMIMTLFLTMVVSMGAVNIRTRSHLAPFCIGLTVTANILAGGGISGACMNPARAFGPAIVSGHWTYHWIYWVGPLTGALVTVSLVSHRVTQCTGWCTMS |
| CcAqp8aa-2 | MTSAESKSELFTVATGDGGDAHQNQPKKLSFYEHYIQPCLAEVLGSTLFMFVGCVSVMGNVGISGSIQPALAHGLALAIAIAIFGEISGGHFNPAVSVCVYLIGGMELMLLVPYVLSQMLGGVIAASLAKAVTTNEAFANATGAAFSAVQTSHGTGSATVAEMIMTLFLTMVVSMGAVNGRTRSQLAPFCIGLTVTANILAGGGISGACMNPARAFGPAIVSGHWEYHWIYWVGPLTGALVTVSLVRLVMGDKKIRVIFK |
| CcAqp8ab-1 | MSAEAEKLELEELEETLLKKNKPKPLGKYERIIQPCVAELVGTTFFVFIGCVSVIENVEAAGRLQPALVHGLAVAVLVACMAEISGSHFNPPFTIAIWLCGGIQLTMVVPYLISQIIGGVLGAAMSKVMTSHENYVNATGAAFTILKSEEQLGKVVFAEMAMTCLVTMVVLLGAVNGKSKSPMVPFMVGCTVIVNVLAGGDISGTCLNPARAFGPALMANYWTYHWVYWVGPIGGGLVAAALVRLLLGDGKIRVILK |
| CcAqp8ab-2 | MSAQAEKLELEELDKTLLKKNEPKPLGKYERIIQPYVAELHPDDYNLHLCMDWQWLCWWHVWQKSACGRCAGSCNVKGVNKYALITHTEYNIVTSRNPKHYSTTDKYILKIMTRENYMNATGATFSVLKSDEQLGKAVFAEMAMTCLVTMVVLLGAVNGKSKSPLV |
| CcAqp8bb | MAEDKVELHNMEKALMDKMGQHDVRESNLFERLFQPCVAELVGTAFFVFIGCVSVIENVSDAGRLQPALAHGLAVAVLIACMAEISGSHFNPSFTIAVYLCGGMELKMVAPYLISQLTGGLLGAVMANGMTSNEKYAQAQGAAFTVLQADDHVWKPLFGEIAMTCLVTMVVLLGAVNAKSKSPLVPFLVGCTVIINVLVGGDVSGTCLNPARALGPAVLTNYWTHHWVYWVGPITGGLIAAALVRILLGDNNTRLLMK |
| CcAqp9a-1 | MKHHCAIKHGIFKEFLAEFLGTFVLVLFGCGSVAQTVLSRNTLGEPLTVHIGFSTGLMMGVYVAGGVSGGHLNPAVSLAMVILGKLKIWKFPVYVTAQMLGAFVGAAAVFGLYYDAFMDFTSGILSVTGINATGHIFASYPGRHLTVLGGFVDQVVGTGMLVLCILAVVDGRNMGAPRGMEPLAVGLILLGISVSMGLNCGYPLNPARDLGPRLFTAAAGWGMEVFSTADYWWWIPVAGPLVGGVAGAVIYFLLIELHHSNHNDKSHKEPEEEVEEEDEDSSLKDKYEMINMS |
| CcAqp9a-2 | MKHHCAIKHGIFKEFLAEFLGTFVLVLFGCGSVAQTVLSRNTLGEPLTVHIGFSTGLMMGVYVAGGVSGGHLNPAVSLAMVILGKLKIWKFPIYVTAQMLGAFVGAAAVFGLYYDAFMDFTSGILSVTGINATGHIFASYPGRHLTVLGGFVDQVVGTGILVLCILAVVDGRNIGAPRGVEPLAVGLILLGISVSMGLNCGYPLNPARDLGPRLFTAAAGWGMEVFSTADYWWWIPVAGPLVGGVAGAVIYFLLIELHHTNHSDKPHEEPEEEEEEDEDEDEDSSLKDKYEMINMS |
| CcAqp9b-1 | MELENFRNLRERCTLRRDIIREFLAELLGTFVLILFGCGSVAQTILSREKQGENLTIHFGFTLGVMLAVYMAGGVSGGHVNPAVSLAMVVLGKLPLKKFPVYVAAQFLGAFAGSCAVFCLYYGAFANFADGKQIVVGENATAGIFASYPREDLSLLNGFIDQVIGTGALVLCILAIVDKKNIGAPKGMEPLVIGLSILAIGVSMALNCGYPINPARDLGPRLFTAMAGWGLEVFRAGNGWWWVPVVGPMVGGVAGAVIYFLMIELHHPELEKNLEDDNSIKDKYELNTVN |
| CcAqp9b-2 | MELEHIRNLRERCTVRRDIIREFLAELLGTFVLILFGCGSVAQTILSREKQGENLTIHFGFTLGVMLAVYMAGGVSGGHVNPAVSLAMVVLGKLPLKKFPVYVVAQFLGAFVGSCAVFCLYYGAFANFADGKRIVNGENATAGIFASYPREDLSLLNGLIDQVIGTGALVLCILAIVDNKNIGAPKGMEPLVIGLSILAIGVSMALNCGYPINPARDLGPRLFTAMAGWGLDVFRAGNGWWWVPVVGPMVGGVLGAVIYFLMIELHHPEPEKNLEDDNSIKDKYELNTVN |
| CcAqp10a-1 | MSRLKKIIKRMKVKNELVRQIMGEVLGTFVLLLFGCAAAAQVKTSRETKGQFLSVNMAFSIGVMSAMYLSRAVSGAHLNPAVSLSFCVLGDLAWIKLLPYSLAQIFGAYLASGLVYLIYHDAIMEFSGGVLTVFGPNETASIFATYPTDVVSMQTSFLDQVVGTAMLMLCILPLNDKRNAPAPEALLPPIVATVVLGISMSMSANCGAAINPARDLGPRLFTFTAGWGIEVFTCYDYFFWIPLVAPMVGGVLGSIIYLVFIQWHLPEPEDESESEEFNEQTKVTEHNDKKDEFFYKMSSL |
| CcAqp10a-2 | MSRIKKVIKRMKVKNELARQIMGEVLGTFVLLLFGCAAAAQVKTSRETKGQFLSVNMAFSIGVMCAMYLCRAVSGAHLNPAVSLSFCVLGDLAWIKLLPYSLAQILGAYLASGLVYFIYHDAIMEFSGGVLTVFGPNETASIFVTYPTDVVSVQTSFLDQVVGTAMLMLCILPLNDKRNAPAPEALLPPIVGTVVLGISMSMSANCGAAINPARDLGPRLFIFTAGWGTEVFTCYDYFFWIPLVAPMVGGVLGSIIYLVFIQWHLPEPEDESDEDSFNEQTKCKAESGKQQMQMQTPPLHPASLILLLSSSWP |
| CcAqp10b-1 | MMDRLLRRCRIKSRLFRECLAEFFGVYILILFGCGSVAQVTTSQNTKGEYLSINLGFALGTTFGIYVAKGVSGAHLNPAVSLTLCVLGRFSWTCLPFYVCSQLLGAFLAAATVALQYYDAIMDFTGGHLTVSGATATAGIFSTYPADYLSLWGGVVDQIIGTAALLVCVLALGDPRNTPAPPGLEPVLVGAAVLVIGISMGSNSGYAINPARDFGPRLFSYIAGWGDEVFRAGHGWWWVPVFVTCVGALLGALLYELLIGVHHPDSEPEEAEDLTAVLQQTVELDGMQPKFDFFKENGKDGIFSITSADTG |
| CcAqp10b-2 | MMDRLLRRCRIKSRLFRECLAEFFGVYILILFGCGSVAQVTTSQNTKGEYLSINLGFALGTTFGIYVAKGVSGAHLNPAVSLTLCVLGRFSWTCLPFYVCSQLLGAFLAAATVALQYYEDFGPLGNSPVLLLLSPDAIMDFTGGHLTVSGATATAGIFSTYPADYLSLWGGVVDQIIGTAALLVCVLALGDPRNTPAPPGLEPVLVGAAVLVIGISMGSNSGYAINPARDFGPRLFSYIAGWGDEVFRAGHGWWWVPVFVTCVGALLGALLYELLIGVHHPDSEPEEAEDLTAVLQQTVELDGMQPKFDFFKENGKDGIFSITSADTG |
| CcAqp11b-1 | MAGMADLTVSLSFLLGIVVFSEVARRTALYLFPNRNWIIYALELISTFQLCACTHELKLLAEIGGLEPRIALTLTFLISVVHGLSFRGAICNPTGALEQLYLGTLMRRCALTRISCQLIAAEAARRVMPHAWALALSDLHAQHSLTGFSCTNSPVNAPLPQAAAVELGCAFVMHTAAFNAEKVEEKYRVPAMAAVITILVYAGGHLTGAVFNPALAFSIQFVCPGNSFAEYSFVYWIGPILGMTGSLLLSDKVIPAISGKSTIPKHSNRLETKKMK |
| CcAqp11b-2 | MAEMADLSVSLSLLLLIVVFSEVARRTALYLFQNRNWIIYVLELISTFQLCACTHELKLLAEVRGLDPQLALTLTYLISVVHGLSFHGAICNPTGTLEQLWHGALTRGCALTRISCQLIAAEAARRVMPHAWALALSDLHAQHSATGFKCASSPVNAPLLQAAAVELSCAFVMHTAVSKVEKVDEKYRVPAIAAVITILVYAGGHLTGAVFNPALAFSVQFPCPGNSFAEYSFVYWIGPVLGMTGSLLLFDKAIPAISGKRTNPKQLNSNGLKVKKMK |
| CcAqp12-1 | MSGLNASLGYFLGIVGVSAVGRVLVTRWRPRWTFLTEFIAAFALSACRLEVDTIAEVGQWAGALGPDVAITMLFISITVHGIIMQGVSGNPSVTLMGLLQKDTGAVAAVLSVAAQLLGAYVALLVTGKYWQMELTDMHMIKNMMMSECSTSLKVPPLQGVFAEALGALTFHMVYLVLKSRSQLLRIPIFAVLLTLIAYAGNNYTSGYVNPSLAYAMTFTCPGHTLLAYSLIYWLGPLIGMTLALFLYLGNIPLLFSRNLLYSKKARFRVPKGKTSEDKTS |
| CcAqp12-2 | MSGLNTSLGYFSAIVGVSAVGRVLFARWTFLTEFIAAFALAASRLEVDTIAEVGQWAGAIGPDFAVTMLFISLAIHGVVMQGVSGNPSVTLMGLLQKEAGAASVVLRVAAQLLGAYVGLLVAGKYWQMELTDMHMIKNLLMSECSTSLQVPALQGVFAEALGALTFHLVYLIMKNRSQLLQIPIFTVLLTFIAYAGNNYTSGYVNPSLAYAMTFTCPGHTFLAYSLVYWLGPLIGMTLALFLYLGNIPLLFSRNLLYSKKPRVWVPKGKTSEDKSS |
| CcAqp14-1 | MAIKEELRSRQFWQGILAETLGSLIFVSAVLGSLVPGPDGASPGPIYPALAAGMATVVLGYCFGEISGAQVNPAVTVALLAMRKVDVLRAVVYLLAQCLGGILAAGLMYLSLPLKSTAQNFINKVPVDMNAGQALGMEMLATFLLSFTVFSVEDQRRRDINEPGNLAIGFAVTTAIFIAGRFSGASLNPARSLGPAIILGYWEHHWVYWIGPILGAVLAGVSHEFIFAPSASRQKLVACLTCKDIEIVETASVSRSSLSTVTQSAMRNKQSNKLEHS |
| CcAqp14-2 | MAIKEELRSRQFWQGILAETLGSLIFVSAVLGSLVPGPDGASPGPIYPALAAGMATVVLGYCFGEISGAQVNPAVTVALLATRKVDVLRAVVYLLAQCLGGILAAGLMYLSLPLKSTAQNYINKVPVEMNAGQALGMEMLATFLLGFTVFSVEDQRRREINEPGNLAIGFAVTTAIFIAGRFSGASLNPARSLGPAIILGYWEHHWVYWIGPIFGAVLAGVSHEFIFAPSASRQKLMACLTCKDIEIVEMASVSRSSLSAVTQSAMRNKQSN |
| CcAqp15-1 | MTHSFTLTGERQAGRQVVTTSEEHMRAFSIKRLNQVRPSSLAPLAVFRKDQASGQRRARIKTANSKFIAKGDLDYKEDQLKKKHHETLVHIQLDPKGWPLSWDSITDEPLPTLDSSLACSPDPLHVSLAFGVSLILTSVCLGEVHLNPVVTLALVAGLRVSPWRAVLLVGAQLLGALSACALLLDITPTALKSNLGVNEMWLMRVGATLDLLISSLHQCSHTLFLERKWGNSKMGMDGWREIESKRERREDNIVCQVAPGVYLYQALLVEMAVTFQLVLCVQAATHPKSAFSSVAPVVIGLSVTLGHLVAIGFTGCGMNPARSFGPAVLTMNFHNHWVYWVGPCAGSLLAWVLHDLVLHPRWSCLGDWVTECKETLLKDLSKQSRGPENHIEA |
| CcAqp15-2 | MGIKSKRERKEEKIVCQVAPGVYLYQALLVEMAVTFQLVLCVQAASQPKSVFSSVAPAVICLSVTHGHIMADTTLYNDMGMTQVYWVGLCSGFLLAWLLHDLVLHPRWRLVDRMCTGWDFVLAFFLPGYCMTWCSILVGDWLTECKEAFLKDLSKQFRGSENHMEA |
